# An Overview of the Focus of the International Gap Junction Conference 2017 and Future Perspectives

**DOI:** 10.3390/ijms19092823

**Published:** 2018-09-18

**Authors:** Patricia E. Martin, Brenda R. Kwak

**Affiliations:** 1 Department of Biological and Biomedical Sciences, School of Health and Life Sciences, Glasgow Caledonian University, Glasgow G4 0BA, UK; 2 Department of Pathology and Immunology, University of Geneva, CH-1211 Geneva, Switzerland

This Special Issue relates to the 18th biannual International Gap Junction Conference (IGJC2017), held at the Crowne Plaza Hotel, Glasgow, U.K., from the 29 July–2 August 2017. The special issue, entitled: Interplay of Connexins and Pannexins in Tissue Function and Disease focused on six key state of the art reviews written by chairs of the sessions highlighting the assembly and functional interactions of connexins and pannexins in diverse tissues and disease states, with translational outputs emerging. A further 14 articles detail specific contributions that were presented as oral communications (Table 1). The meeting was attended by over 200 delegates from 24 different countries and celebrated 50 years of Gap Junction Research.

Connexins and pannexins are tetramembrane spanning channel proteins with a shared topology and related functional properties. They oligomerise to form channels in the plasma membrane with two extracellular loops that project into the extracellular space and an intracellular carboxyl tail is subject to post-translational modification. Within the connexin family (21 members in man), these extracellular loops interdigitate and dock with loops from neighbouring cells to form dodecameric intercellular gap junction channels (GJCs) that link the cytoplasm’s of neighbouring cells. These GJCs enable the regulated exchange of over 300,000 metabolites of less than 1000 Da in size, in so doing co-ordinating specific cell and tissue homeostasis. Connexin compatibility is highlighted by phylogenetic classification into three–four specific subgroups, with only specific heteromeric channel combinations possible and in so doing facilitating the segregation of tissue compartments.

Over the last 15 years, it has emerged that connexin hemichannels can be triggered to open under conditions of cell stress releasing signalling molecules such as ATP, glutamate and Nicotinamide adenine dinucleotide (NAD) into the extracellular environment and subsequently elicit localised extracellular signalling cascades via purinergic receptors. Pannexins, proteins forming hemichannel-like structures, identified about 15 years ago, share a common topology but no sequence homology with connexins, are thought to be evolutionary related to the innexins, gap junction forming proteins in invertebrates. Unlike connexins (and innexins) pannexins are highly glycosylated proteins and act to release ATP, engaging with downstream signalling pathways. In particular, pannexin signalling has been closely linked with inflammatory mediated events, where caspase1 cleavage of the carboxyl tail renders these channels constitutively open and triggers cell death ‘find me’ signals.

Due to the diversity of connexin and pannexin expression in tissue networks, their importance cannot be underestimated and they are now firmly established as key proteins in diverse tissue networks including the cardiovascular system, the skin, the nervous system, the liver, the ocular, respiratory and immune system. Further, changes in connexin expression and function occur in disease states associated with all of these tissues: Tumorigenesis, diabetic retinopathy, cardiac arrhythmia, atherosclerosis, stroke, Alzheimer’s disease, chronic non–healing skin wounds, and inflammation of epithelial tissue, to name a few. A range of mutations in connexins are associated with clinical disease, where mutations in Cx26 are among the most common in recessively inherited hearing impairment, Cx32 with the demyelinating disorder Charcot Marie Tooth-Linked disease, Cx43 with oculodentodigital dysplasia, and Cx46 and Cx50 with familial cataract formation. All these diseases impact on the quality of life, healthcare resources, ageing populations and many with conditions that can be managed with current therapies, but not cured.

Thus, connexins and pannexins have emerged as prime therapeutic targets for a diverse range of disease states. These channels are amenable to targeting therapeutically and a range of antisense and peptidomimetic strategies have emerged as key regulators of channel function. Such regulators were first identified over 25 years ago when Evans and Warner synthesised the first mimetic peptides and antibodies that mimicked amino acid sequences on the extracellular loops. These tools are now widely used by the research community to define the role of connexins and pannexins in tissue function and are proving successful in translational research where the success of clinical trials of a connexin targeted therapy, from bench to bedside, for improving wound healing events is reported in this special issue.

The Editors thank all contributors to this special issue, delegates and in the conference support team for the successful running of IGJC2017. The follow-up meeting will be held in Victoria, Vancouver Island, July 2019 (#IGJC2019).

## 1. Contents of the Special Issue

### 1.1. Trafficking, Assembly, Gating and Protein-Protein Interactions

**Reviews**

- Aasen, T.; Johnstone, S.; Vidal-Brime, L.; Lynn, K.S.; Koval, M. Connexins: Synthesis, Post-Translational Modifications, and Trafficking in Health and Disease. *Int. J. Mol. Sci.* **2018**, 19(5). [1]
- Sorgen, P.L.; Trease, A.J.; Spagnol, G.; Delmar, M.; Nielsen, M.S. Protein–Protein Interactions with Connexin 43: Regulation and Function. *Int. J. Mol. Sci.* **2018**, 19(5). [2]

**Participant Contributions**

- Ek-Vitorin, J.F.; Pontifex, T.K.; Burt, J.M. Cx43 Channel Gating and Permeation: Multiple Phosphorylation-Dependent Roles of the Carboxyl Terminus. *Int. J. Mol. Sci.* **2018**, 19(6). [3]
- Spagnol, G.; Trease, A.J.; Zheng, L.; Gutierrez, M.; Bazu, I.; Sarmiento, C.; Moore, G.; Cervantes, M.; Sorgen, P.L. Connexin43 Carboxyl-Terminal Domain Directly Interacts with β-Catenin. *Int. J. Mol. Sci.* **2018**, 19(6). [4]
- Sanchez-Pupo, R.E.; Johnston, D.; Penuela, S. N-Glycosylation Regulates Pannexin 2 Localization but Is Not Required for Interacting with Pannexin 1. *Int. J. Mol. Sci.* **2018**, 19(7). [5]
- Batissoco, A.C.; Salazar-Silva, R.; Oiticica, J.; Bento, R.F.; Mingroni-Netto, R.C.; Haddad, L.A. A Cell Junctional Protein Network Associated with Connexin-26. *Int. J. Mol. Sci.* **2018**, 19(9). [6]
- Schadzek, P.; Helmes, D.; Stahl, Y.; Dilger, N.; Ngezahayo, A. Concatenation of human connexin26 (hCx26) and human connexin46 (hCx46) for the analysis of heteromeric gap junction hemichannels and heterotypic gap junction channels. **2018**, 19(9). [7]

### 1.2. Cardiovascular System

**Review**

- Molica, F.; Figueroa, X.F.; Kwak, B.R.; Isakson, B.E.; Gibbins, J.M. Connexins and Pannexins in Vascular Function and Disease. *Int. J. Mol. Sci.* **2018**, 19(6). [8]

**Participant Contributions**

- Carballo, S.; Pfenniger, A.; Carballo, D.; Garin, N.; James, R.W.; Mach, F.; Shah, D.; Kwak, B.R. Differential Association of Cx37 and Cx40 Genetic Variants in Atrial Fibrillation with and without Underlying Structural Heart Disease. *Int. J. Mol. Sci.* **2018**, 19(1). [9]
- Noureldin, M.; Chen H, Bai D. Functional Characterization of Novel Atrial Fibrillation-Linked GJA5 (Cx40) Mutants. *Int. J. Mol. Sci.* **2018**, 19(4). [10]
- Viczenczova, C.; Kura, B.; Egan Benova, T.; Yin, C.; Kukreja, R.C.; Slezak, J.; Tribulova, N.; Szeiffova Bacova, B. Irradiation-Induced Cardiac Connexin-43 and miR-21 Responses Are Hampered by Treatment with Atorvastatin and Aspirin. *Int. J. Mol. Sci.* **2018**, 19(4). [11]
- Boucher, J.; Simonneau, C.; Denet, G.; Clarhaut, J.; Balandre, A.C.; Mesnil, M.; Cronier, L; Monvoisin, A. Pannexin-1 in Human Lymphatic Endothelial Cells Regulates Lymphangiogenesis. *Int. J. Mol. Sci.* **2018**, 19(6) [12]
- Htet M, Nally, J.E.; Shaw, A.; Foote, B.E.; Martin, P.E.; Dempsie, Y. Connexin 43 Plays a Role in Pulmonary Vascular Reactivity in Mice. *Int. J. Mol. Sci.* **2018**, 19(7). [13]

### 1.3. Connexins and Tumorigenesis

**Review**

- Graham, S.V.; Jiang, J.X.; Mesnil, M. Connexins and Pannexins: Important Players in Tumorigenesis, Metastasis and Potential Therapeutics. *Int. J. Mol. Sci.* **2018**, 19(6). [14]

**Participant Contributions**

- Busby, M.; Hallett, M.T.; Plante, I. The Complex Subtype-Dependent Role of Connexin 43 (GJA1) in Breast Cancer. *Int. J. Mol. Sci.* **2018**, 19(3). [15]
- Iikawa, N.; Yamamoto, Y.; Kawasaki, Y.; Nishijima-Matsunobu, A.; Suzuki, M.; Yamada, T.; Omori, Y. Intrinsic Oncogenic Function of Intracellular Connexin26 Protein in Head and Neck Squamous Cell Carcinoma Cells. *Int. J. Mol. Sci.* **2018**, 19(7). [16]

### 1.4. Epithelial Tissue, Wound Healing

**Reviews**

- Chanson, M.; Watanabe, M.; O’Shaughnessy, E.M.; Zoso, A.; Martin, P.E. Connexin Communication Compartments and Wound Repair in Epithelial Tissue. *Int. J. Mol. Sci.* **2018**, 19(5). [17]

**Participant Contributions**

- Faniku, C.; O’Shaughnessy, E.; Lorraine, C.; Johnstone, S.R.; Graham, A.; Greenhough, S.; Martin, P.E. The Connexin Mimetic Peptide Gap27 and Cx43-Knockdown Reveal Differential Roles for Connexin43 in Wound Closure Events in Skin Model Systems. *Int. J. Mol. Sci.* **2018**, 19(2). [18]
- Chen, J.; Liang, C.; Zong, L.; Zhu, Y.; Zhao, H.B. Knockout of Pannexin-1 Induces Hearing Loss. *Int. J. Mol. Sci.* **2018**, 19(5). [19]

### 1.5. Connexin Therapy Translates to Clinic

**Review**

- Montgomery, J.; Ghatnekar, G.S.; Grek, C.L.; Moyer, K.E.; Gourdie, R.G. Connexin 43-Based Therapeutics for Dermal Wound Healing. *Int. J. Mol. Sci.* **2018**, 19(6). [20]

## Figures and Tables

**Table 1:** Publication contents of this special issue.

Topic	Reviews	Participant Contributions
Trafficking, Assembly, Gating and Protein-Protein Interactions	[1,2]	[3,4,5,6,7]
Cardiovascular System	[8]	[9,10,11,12,13]
Connexins and Tumorigenesis	[14]	[15,16]
Epithelial Tissue, Wound Healing	[17]	[18,19]
Connexin Therapy Translates to Clinic	[20]	
